# Identifying Factors Associated With Patient Portal and Synchronous Telehealth Use Across Age Groups in the Postpandemic Era: Retrospective Analysis of the Health Information National Trends Survey

**DOI:** 10.2196/83730

**Published:** 2026-03-03

**Authors:** Jaeyoung Park, Su-I Hou

**Affiliations:** 1 School of Global Health Management and Informatics, University of Central Florida, 528 W Livingston St, Orlando, FL, 32801, United States, 1 407-823-3344

**Keywords:** telehealth, eHealth, mHealth, virtual healthcare, health services for the aged

## Abstract

**Background:**

Since the COVID-19 pandemic, telehealth has become a core component of modern health care, encompassing both synchronous services (real-time video or phone) and patient portals, which offer a wide range of online features to streamline care delivery, including asynchronous communication and access to medical records. Older adults often face greater barriers to these technologies, potentially widening health care access gaps.

**Objective:**

This study examines factors influencing the adoption of patient portals and synchronous telehealth in the postpandemic era. It compares the 2 modalities and identifies distinct usage patterns across age groups to inform targeted strategies.

**Methods:**

We analyzed data from the Health Information National Trends Survey (HINTS) Cycles 6 and 7. Outcomes over the past 12 months included (1) frequency of patient portal use (continuous), (2) use of synchronous telehealth (binary), and (3) no engagement with any telehealth services (binary). Key variables included demographics, socioeconomic status, technology familiarity, and health care behaviors. Age groups were categorized as young (18–49 years), middle-aged (50–64 years), and older adults (≥65 years). Interaction terms between age groups and other variables were included to uncover age-specific patterns. To mitigate multicollinearity arising from the interaction terms, we used penalized linear and logistic regression models using the least absolute shrinkage and selection operator (LASSO) penalty without survey weights, supplemented by bootstrapping to evaluate the stability. Using the selected variables, we then fitted survey-weighted generalized linear models to account for the complex design of the HINTS survey and more accurately represent the US population.

**Results:**

Among 12,865 respondents, 36.1% (n=4638) were 65 years or older. Overall, 65.2% used patient portals and 38.4% used synchronous telehealth. Age alone was not a significant factor, but older adults exhibited distinct patterns. Older adults with higher care frequency were less likely to use patient portals (coefficient −0.07, 95% CI −0.10 to −0.04; *P*<.001). Those who completed the survey online in this age group were more likely to use patient portals (coefficient 0.32, 95% CI 0.18-0.47; *P*<.001) than to use a paper form. Older Black or African American patients were less likely to use patient portals (coefficient −0.30, 95% CI −0.45 to −0.16; *P*<.001). These age-specific patterns were identified only in patient portal use, not in synchronous telehealth use.

**Conclusions:**

Telehealth usage among older adults differs in subtle ways from other age groups, and the factors driving adoption vary between patient portals and synchronous care. These findings underscore the need for age- and context-specific strategies to enhance the uptake of both types of services.

## Introduction

Telehealth has emerged as a cost-effective and integral component of modern health care, offering both patient portals and synchronous care (eg, real-time video or phone consultations) to improve access to care [1]. Synchronous telehealth facilitates live communication with health care providers without the need for long-distance travel, while patient portals streamline care delivery and reduce unnecessary office visits through various features such as asynchronous communications, access to medical records, medication refills, and appointment scheduling.

As the demand for remote health care continues to grow, telehealth has expanded beyond primary care into numerous specialties, with many providers now offering both in-person and virtual care options [2]. Despite its many advantages, telehealth has also introduced new challenges, particularly in exacerbating existing disparities in health care access [3]. For instance, individuals living in areas with limited internet infrastructure have struggled to benefit from telehealth services [4,5]. Moreover, the digital divide, driven by gaps in digital literacy, has hindered telehealth adoption, especially among older adults [5,6]. Additional barriers such as limited health literacy [7] and English proficiency [8] further complicate access and use.

Although research on telehealth is expanding, critical gaps remain. The barriers to telehealth use may be intricately linked to the modality itself, with patient portal and synchronous services presenting distinct challenges and opportunities. This suggests that tailored strategies may be necessary to enhance use across different modalities. Furthermore, patterns of telehealth use may vary significantly by age group, indicating the need for age-specific approaches to promote equitable access.

Using survey data collected in the postpandemic period, this study investigates the complex relationships between 2 types of telehealth modalities (ie, patient portals and synchronous telehealth) and various patient factors, including demographic factors, socioeconomic status, medical conditions, care behaviors, and technological proficiency. For the development of effective telehealth interventions, we compare the key drivers of use across modalities. Additionally, we explore how these factors differ among age groups, particularly among older adults, testing the hypothesis that telehealth usage patterns are uniquely shaped by age-related characteristics.

## Methods

### Data

In this study, we used the Health Information National Trends Survey (HINTS) Cycles 6 and 7, where the surveys were conducted in 2022 and 2024, respectively [9]. These surveys include several categories of questions, such as, “Looking for Health Information, Internet and Technology Use” (in Cycle 7; referred to as “Using the Internet to Find Information” in Cycle 6), “Your Health Care,” “Telehealth,” and “You and Your Household.” Assuming no period effects, as all the survey periods were conducted postpandemic, we harmonized the 2 datasets without accounting for the survey cycles. We used only overlapping variables with necessary name and type adjustments (see Table S1 in Multimedia Appendix 1) and combined the full sample weight and 50 replicate weights following HINTS guidelines [10]. Details on data preparation are available in the GitHub repository [11].

### Outcomes

To identify the use of patient portals and synchronous telehealth, we used the following survey questions: “How many times did you access your online medical record or patient portal in the last 12 months?” (AccessOnlineRecord2 in Cycle 6 and AccessOnlineRecord3 in Cycle 7) for patient portal usage, and “In the past 12 months, did you receive care from a doctor or health professional using telehealth?” (ReceiveTelehealthCare) for synchronous telehealth usage. The patient portal question captures usage frequency, with 5 response options, including 0, 1‐2, 3‐5, 6‐9, and 10 or more times. Patients who selected “I do not have an online medical record or patient portal” were classified as having no use (ie, 0 times). For simplicity in implementing parametric models, this outcome was treated as continuous in the model [12], with the 5 response categories coded from 0 to 4. On the other hand, the question about synchronous telehealth focuses on the method of experience, with possible answers being no, yes by video, yes by phone call, and yes by both. For analysis, all “yes” responses were combined and treated as a binary outcome. Additionally, we created a dichotomous variable “Neither” to indicate patients who had not used any form of telehealth, meaning neither patient portals nor synchronous telehealth. We excluded those who did not answer at least one of the telehealth questions (ie, AccessOnlineRecord2/3 or ReceiveTelehealthCare) to ensure consistent analytic samples across all outcomes.

### Variables

Based on existing studies [5,6,13-15], variables included demographic and socioeconomic status information (ie, age, race, ethnicity, birth sex, total number of households, and residence), tech-savviness (ie, internet use and social media use), surveys, and care behaviors (ie, frequency of care services, ability to take good care of health). The 5 age categories in the data (ie, 18‐34 years, 35‐49 years, 50‐64 years, 65‐74 years, and ≥75 years) were combined into the following 3 groups, including young (18-49 years), middle (50-64 years), and old (≥65 years). Missing values for the independent variables were imputed using multivariate imputation by chained equations (MICE) [16] without survey weights. This imputation process included all independent variables and both dependent variables (patient portal and synchronous telehealth use) and produced converged imputed values after 5 iterations. Table S1 in Multimedia Appendix 1 presents the missing rates.

### Models

To examine how the 3 outcomes (ie, patient portals*,* synchronous, and Neither) were associated with various variables across age groups, our models incorporated interaction terms between age groups and the other variables. The 3 models used the same analytic cohort and the same set of independent variables. For the patient portal and synchronous telehealth models, use of the other telehealth modality was also added to the set of independent variables (ie, synchronous telehealth uses in the patient portal model, and vice versa). To facilitate straightforward interpretation, ordinal independent variables, in addition to the patient portal frequency outcome, were treated as continuous in the models [12].

We used a two-step approach: (1) variable selection using unweighted penalized regression models and (2) statistical inference with survey-weighted generalized linear models. As the first step, we selected variables to prevent multicollinearity and overfitting from interaction terms. This screening process was conducted solely for model specification and did not involve the use of survey weights. We built penalized linear or logistic regression models with the least absolute shrinkage and selection operator (LASSO) penalty for each outcome [17]. We applied LASSO linear regression for the patient portal outcome and LASSO logistic regression for the synchronous telehealth and Neither outcomes. The LASSO hyperparameter, penalty magnitude (λ), was determined using 5-fold cross-validation and selected based on the penalty value that produced the smallest average error across the 5 validation sets. To assess stability, we bootstrapped 500 samples, fitted penalized linear models with LASSO, and calculated the selection frequency, 95% CIs, and *P* values for each variable based on *t* tests, defined as the mean divided by the SD [18]. Variables with a *P* value of less than .05 were selected.

In the second stage, we fitted survey-weighted generalized linear models using the selected variables to account for the complex design of the HINTS survey and ensure accurate representation of the US population [19]. We used the full sample weight to produce population-level estimates and the replicate weights to calculate the standard error and variance of those estimates. For model evaluation, adjusted *R*^2^ (for the patient portal model) and pseudo-*R*^2^ (for the synchronous and Neither models) were used to assess goodness of fit [20,21]. In human subject research, these values between 0.10 and 0.30 are generally considered acceptable [21,22]. We reported coefficients for linear regression, odds ratio (OR) for logistic regression, and 95% CI and *P* value for both. For inference, a positive coefficient indicates being more likely to use patient portals, while an OR over 1 indicates being more likely to use synchronous telehealth in the synchronous model or never use any form of telehealth in the Neither model. The variable significance was determined when the variable’s *P* value was smaller than the significance level (.05).

All analyses were conducted using R programming (version 4.4.3; R Core Team) with the following packages: *forestplot* [23], *glmnet* [17,24], *mice* [16], and *survey* [19].

### Sensitivity Analyses

We conducted sensitivity analyses under 3 different scenarios to assess potential bias arising from data cleaning. First, to assess potential bias introduced by imputation, we created 5 different imputed datasets using the same imputation method (ie, MICE) with different random seeds. Models were then built on each imputed dataset, and significant variables were compared across them. Second, since survey form type may reflect technology familiarity, we excluded this variable from the entire analysis (from imputing missing values through constructing models) and then investigated changes in the identifications of significant factors among other technology familiarity variables (ie, social media interaction and internet use). Finally, since we treated ordinal patient portal use as continuous, we applied an additional modification to confirm its reasonableness, categorizing portal use into 2 groups (<3 and ≥3 times), treating it as binary, and rerunning the entire analysis for this outcome. This new outcome type required a different regression model than the original, making the results not directly comparable in size or magnitude. Instead, we focused on whether the same variables were significant and whether their effects pointed in the same direction (positive or negative).

### Ethical Considerations

This study used publicly available, deidentified HINTS data to conduct a secondary analysis. The dataset contains no personal identifiers. Because the dataset is publicly available and deidentified, this research does not constitute human subjects research as defined by federal regulations (45 CFR 46.102) and is therefore exempt from institutional ethics review. Accordingly, institutional review board review at the authors’ institution was not required.

## Results

### Data

Excluding missing responses to either patient portal or synchronous telehealth usage, we identified 12,865 patients in this study. They were comprised of 4714 (36.6%) young-age (18-49 years), 3513 (27.3%) middle-age (50-64 years), and 4638 (36.1%) old-age (≥65 years) patients. Non-Hispanic White patients accounted for more than half (7204/12,865, 56%), followed by Hispanic (2519/12,865, 19.6%), Non-Hispanic Black or African American (1979/12,865, 15.4%), and Non-Hispanic Asian (680/12,865, 5.3%). More female patients (7739/12,865, 60.2%) participated in this survey than male (5126/12,865, 39.8%). Additionally, 37.7% of patients (4854/12,865) resided in large metropolitan areas and 22.0% (2828/12,865) in large fringe metropolitan areas, while 13.4 % lived in nonmetropolitan areas (1730/12,865). Table 1 provides an overview of the unweighted distributions by telehealth modalities, based on the imputed dataset.

**Table 1.** Variables in this study and their unweighted distributions in total and by the outcomes (N=12,865).

Variables	Total, n (%)	Patient portals					Synchronous	
		0 times, n (%)	1-2 times, n (%)	3-5 times, n (%)	6-9 times, n (%)	≥10 times, n (%)	No use, n (%)	Use, n (%)
Telehealth use								
Demographic and socioeconomic status, n (%)	12,865 (100)	4482 (34.8)	2590 (20.1)	2605 (20.2)	1382 (10.7)	1806 (14.0)	7923 (61.6)	4942 (38.4)
Age (years), n (%)								
18-49	4714 (36.6)	1339 (28.4)	1168 (24.8)	1002 (21.3)	516 (10.9)	689 (14.6)	2721 (57.7)	1993 (42.3)
50-64	3513 (27.3)	1136 (32.3)	682 (19.4)	746 (21.2)	410 (11.7)	539 (15.3)	2138 (60.9)	1375 (39.1)
≥65	4638 (36.1)	2007 (43.3)	740 (16.0)	857 (18.5)	456 (9.8)	578 (12.5)	3064 (66.1)	1574 (33.9)
Sex, n (%)								
Female	7739 (60.2)	2433 (31.4)	1567 (20.2)	1604 (20.7)	908 (11.7)	1227 (15.9)	4524 (58.5)	3215 (41.5)
Male	5126 (39.8)	2049 (40.0)	1023 (20.0)	1001 (19.5)	474 (9.2)	579 (11.3)	3399 (66.3)	1727 (33.7)
Race and ethnicity, n (%)								
Non-Hispanic White	7204 (560)	2163 (30.0)	1405 (19.5)	1562 (21.7)	872 (12.1)	1202 (16.7)	4517 (62.7)	2687 (37.3)
Non-Hispanic Black or African American	1979 (15.4)	799 (40.4)	387 (19.6)	377 (19.1)	189 (9.6)	227 (11.5)	1216 (61.4)	763 (38.6)
Hispanic	2519 (19.6)	1151 (45.7)	531 (21.1)	409 (16.2)	197 (7.8)	231 (9.2)	1482 (58.8)	1037 (41.2)
Non-Hispanic Asian	680 (5.3)	197 (29.0)	167 (24.6)	165 (24.3)	70 (10.3)	81 (11.9)	426 (62.6)	254 (37.4)
Non-Hispanic other	483 (3.8)	172 (35.6)	100 (20.7)	92 (19.0)	54 (11.2)	65 (13.5)	282 (58.4)	201 (41.6)
Total household, n (%)								
1	3893 (30.3)	1718 (44.1)	651 (16.7)	686 (17.6)	354 (9.1)	484 (12.4)	2504 (64.3)	1389 (35.7)
>2	8972 (69.7)	2764 (30.8)	1939 (21.6)	1919 (21.4)	1028 (11.5)	1322 (14.7)	5419 (60.4)	3553 (39.6)
Education, n (%)								
College graduate or more	6168 (47.9)	1361 (22.1)	1367 (22.2)	1507 (24.4)	814 (13.2)	1119 (18.1)	3537 (57.3)	2631 (42.7)
Less than high school	797 (6.2)	545 (68.4)	120 (15.1)	72 (9.0)	34 (4.3)	26 (3.3)	548 (68.8)	249 (31.2)
High school graduate	2202 (17.1)	1199 (54.5)	367 (16.7)	299 (13.6)	165 (7.5)	172 (7.8)	1531 (69.5)	671 (30.5)
Some college	3698 (28.7)	1377 (37.2)	736 (19.9)	727 (19.7)	369 (10.0)	489 (13.2)	2307 (62.4)	1391 (37.6)
Neighborhood, n (%)								
Large metro	4854 (37.7)	1597 (32.9)	1009 (20.8)	991 (20.4)	535 (11.0)	722 (14.9)	2773 (57.1)	2081 (42.9)
Large fringe metro	2828 (22.0)	778 (27.5)	616 (21.8)	689 (24.4)	322 (11.4)	423 (15.0)	1682 (59.5)	1146 (40.5)
Medium small metro	3453 (26.8)	1354 (39.2)	683 (19.8)	608 (17.6)	364 (10.5)	444 (12.9)	2252 (65.2)	1201 (34.8)
Nonmetro	1730 (13.4)	753 (43.5)	282 (16.3)	317 (18.3)	161 (9.3)	217 (12.5)	1216 (70.3)	514 (29.7)
Medical condition								
Depression, n (%)								
No	9430 (73.3)	3492 (37.0)	2006 (21.3)	1909 (20.2)	924 (9.8)	1099 (11.7)	6351 (67.3)	3079 (32.7)
Yes	3435 (26.7)	990 (28.8)	584 (17.0)	696 (20.3)	458 (13.3)	707 (20.6)	1572 (45.8)	1863 (54.2)
Diabetes, n (%)								
No	10,066 (78.2)	3405 (33.8)	2148 (21.3)	2057 (20.4)	1070 (10.6)	1386 (13.8)	6362 (63.2)	3704 (36.8)
Yes	2799 (21.8)	1077 (38.5)	442 (15.8)	548 (19.6)	312 (11.1)	420 (15.0)	1561 (55.8)	1238 (44.2)
High blood pressure, n (%)								
No	7266 (56.5)	2388 (32.9)	1655 (22.8)	1484 (20.4)	764 (10.5)	975 (13.4)	4537 (62.4)	2729 (37.6)
Yes	5599 (43.5)	2094 (37.4)	935 (16.7)	1121 (20.0)	618 (11.0)	831 (14.8)	3386 (60.5)	2213 (39.5)
Heart condition, n (%)								
No	11,552 (89.8)	3946 (34.2)	2418 (20.9)	2365 (20.5)	1247 (10.8)	1576 (13.6)	7165 (62.0)	4387 (38.0)
Yes	1313 (10.2)	536 (40.8)	172 (13.1)	240 (18.3)	135 (10.3)	230 (17.5)	758 (57.7)	555 (42.3)
Lung disease, n (%)								
No	11,147 (86.6)	3942 (35.4)	2298 (20.6)	2270 (20.4)	1185 (10.6)	1452 (13.0)	7070 (63.4)	4077 (36.6)
Yes	1718 (13.4)	540 (31.4)	292 (17.0)	335 (19.5)	197 (11.5)	354 (20.6)	853 (49.7)	865 (50.3)
Tech savviness								
Social media interact, n (%)								
Never	9895 (76.9)	3861 (39.0)	1939 (19.6)	1915 (19.4)	966 (9.8)	1214 (12.3)	6508 (65.8)	3387 (34.2)
Less than once a month	1700 (13.2)	367 (21.6)	371 (21.8)	400 (23.5)	243 (14.3)	319 (18.8)	897 (52.8)	803 (47.2)
A few times a month	710 (5.5)	138 (19.4)	160 (22.5)	169 (23.8)	104 (14.6)	139 (19.6)	305 (43.0)	405 (57.0)
At least once a week or more	560 (4.4)	116 (20.7)	120 (21.4)	121 (21.6)	69 (12.3)	134 (23.9)	213 (38.0)	347 (62.0)
Use internet, n (%)								
No	1240 (9.6)	1014 (81.8)	99 (8.0)	71 (5.7)	27 (2.2)	29 (2.3)	883 (71.2)	357 (28.8)
Yes	11,625 (90.4)	3468 (29.8)	2491 (21.4)	2534 (21.8)	1355 (11.7)	1777 (15.3)	7040 (60.6)	4585 (39.4)
Survey								
Form type, n (%)								
Paper	4686 (36.4)	2528 (53.9)	706 (15.1)	731 (15.6)	343 (7.3)	378 (8.1)	3181 (67.9)	1505 (32.1)
Web	8179 (63.6)	1954 (23.9)	1884 (23.0)	1874 (22.9)	1039 (12.7)	1428 (17.5)	4742 (58.0)	3437 (42.0)
Care behavior								
Self-care ability, n (%)								
Not confident at all or a little	749 (5.8)	334 (44.6)	129 (17.2)	111 (14.8)	75 (10.0)	100 (13.4)	411 (54.9)	338 (45.1)
Somewhat	3149 (24.5)	1191 (37.8)	614 (19.5)	601 (19.1)	319 (10.1)	424 (13.5)	1900 (60.3)	1249 (39.7)
Completely	3307 (25.7)	1126 (34.0)	709 (21.4)	643 (19.4)	365 (11.0)	464 (14.0)	2123 (64.2)	1184 (35.8)
Very	5660 (44.0)	1831 (32.3)	1138 (20.1)	1250 (22.1)	623 (11.0)	818 (14.5)	3489 (61.6)	2171 (38.4)
Care frequency, n (%)								
None	1459 (11.3)	1018 (69.8)	249 (17.1)	106 (7.3)	35 (2.4)	51 (3.5)	1219 (83.6)	240 (16.4)
1 time	1687 (13.1)	710 (42.1)	569 (33.7)	267 (15.8)	80 (4.7)	61 (3.6)	1221 (72.4)	466 (27.6)
2 times	2329 (18.1)	808 (34.7)	630 (27.1)	553 (23.7)	193 (8.3)	145 (6.2)	1544 (66.3)	785 (33.7)
3 times	2015 (15.7)	583 (28.9)	430 (21.3)	560 (27.8)	252 (12.5)	190 (9.4)	1184 (58.8)	831 (41.2)
4 times	1854 (14.4)	535 (28.9)	336 (18.1)	455 (24.5)	284 (15.3)	244 (13.2)	1078 (58.1)	776 (41.9)
5-9 times	2236 (17.4)	510 (22.8)	266 (11.9)	471 (21.1)	396 (17.7)	593 (26.5)	1145 (51.2)	1091 (48.8)
10 or more times	1285 (10.0)	318 (24.7)	110 (8.6)	193 (15.0)	142 (11.1)	522 (40.6)	532 (41.4)	753 (58.6)

Within this cohort, 8383 (65.2%) and 4942 (38.4%) used patient portals and synchronous telehealth at least once in the past 12 months, respectively. Patients who used patient portals used synchronous telehealth more, and vice versa. For example, of the 4942 patients who used synchronous telehealth, 3877 (78.5%) also used patient portals at least once. Moreover, the likelihood of using synchronous telehealth increased progressively with higher levels of patient portal use. Compared to those who never used patient portals, individuals who used it at least 10 times were significantly more likely to use synchronous telehealth, with an OR of 4.9 (See Table 2).

**Table 2.** Unweighted joint distributions of patient portal and synchronous telehealth use.

Variables	Total, n (%)	Patient portals				
		0 time	1-2 times	3-5 times	6-9 times	≥10 times
Telehealth use, n (%)	12865 ( 100)	4482 (34.8)	2590 (20.1)	605 (20.2)	1382 (10.7)	1806 (14.0)
Synchronous, n (%)						
No	7923 (61.6)	3417 (43.1)	1665 (21.0)	1453 (18.3)	674 (8.5)	714 (9.0)
Yes	4942 (38.4)	1065 (21.5)	925 (18.7)	1152 (23.3)	708 (14.3)	1092 (22.1)
Odds ratio (95% CI)	—^a^	reference	1.8 (1.6-2.0)	2.5 (2.3-2.8)	3.4 (3.0-3.8)	4.9 (4.4-5.5)

a Not applicable.

By age group, younger participants were more likely to use both patient portals and synchronous telehealth services (see age rows in Table 1). Among those aged 18‐49 years (n=4714), 3375 (71.6%) participants used patient portals at least once, compared to 2377 (67.7%) in the 50‐64 years age group (n=3513) and 2631 (56.7%) among those aged 65 years and older (n=4638). Similarly, synchronous telehealth was used by 1993 (42.3%) participants in the youngest group, 1375 (39.1%) in the middle-aged group, and 1574 (33.9%) in the oldest group. Furthermore, the mode of synchronous telehealth varied by age. Among those who used synchronous telehealth, use of phone calls only (without video) was significantly more common in the older group (672/1574, 42.7%) than in the younger group (547/1993, 27.4%) and the middle-aged group (423/1375, 30.8%; *P*<.001; *χ*^2^; see Table 3).

**Table 3.** Synchronous telehealth modes by age groups among those who used synchronous telehealth.

Variables	Total	Synchronous telehealth mode, n (%)		
		By video, n (%)	By phone call (voice only with no video), n (%)	Some by video and some by phone call, n (%)
Age (years), n (%)	4942 (100)	2248 (45.5)	1642 (33.2)	1052 (21.3)
18‐49	1993 (42.3)	1000 (50.2)	547 (27.4)	446 (22.4)
50‐64	1375 (39.1)	671 (48.8)	423 (30.8)	281 (20.4)
≥65	1574 (33.9)	577 (36.7)	672 (42.7)	325 (20.6)

### Two-Step Approach for Variable Screening and Model Inference

Unweighted penalized regression models with bootstrapping were applied to select variables: 21 for the patient portal model, 13 for the synchronous model, and 20 for the Neither model, as shown in Tables S2-S4 in Multimedia Appendix 1. Many interaction terms were not selected because the age-specific subgroups had smaller sample sizes. Even though variables within the subgroups were consistently selected more than 480 out of 500 times, their *P* value was sometimes larger than .05 due to the sample sizes (eg, survey type for the middle-aged group in the patient portal model). Estimates, confidence intervals, *P* values, and selection frequencies for all variables are reported in Table S2-S4 in Multimedia Appendix 1.

**Figure 1.**
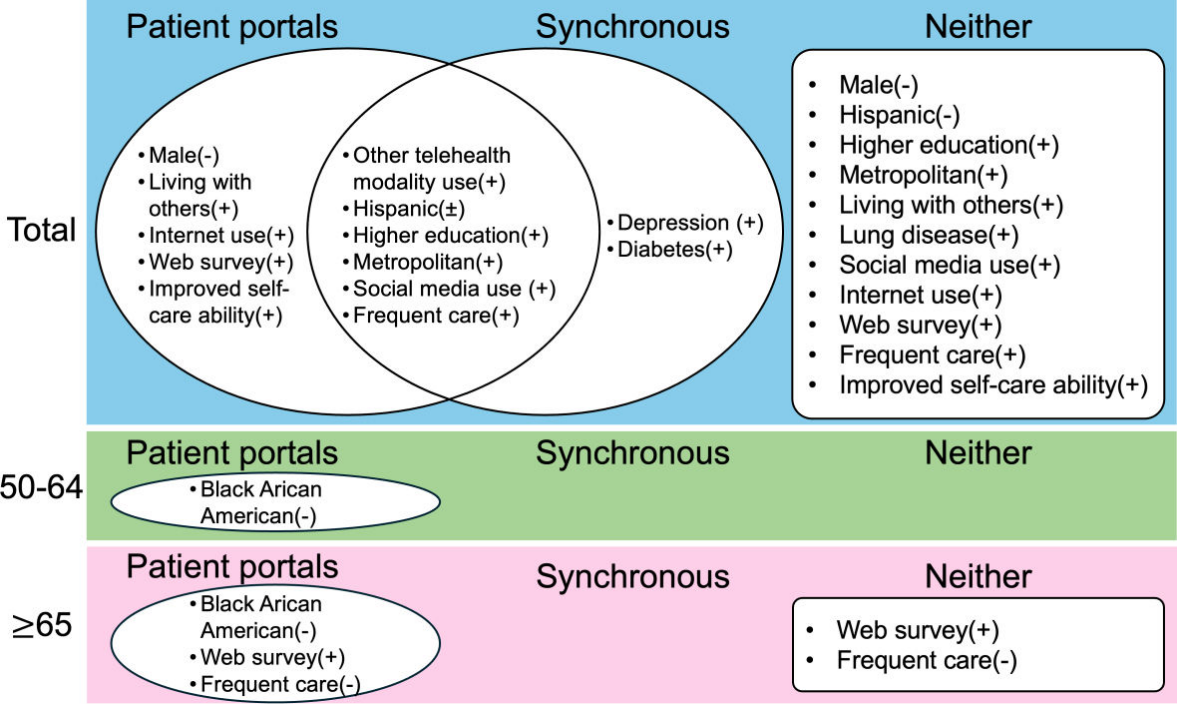
Shared and unique significant variables associated with patient portal and synchronous telehealth use, both overall and across different age groups. The figure also highlights significant variables identified in the Neither model. A plus (+) symbol indicates greater telehealth use, a minus (−) indicates less use, and a plus or minus (±) symbol denotes mixed directions of association.

**Figure 2.**
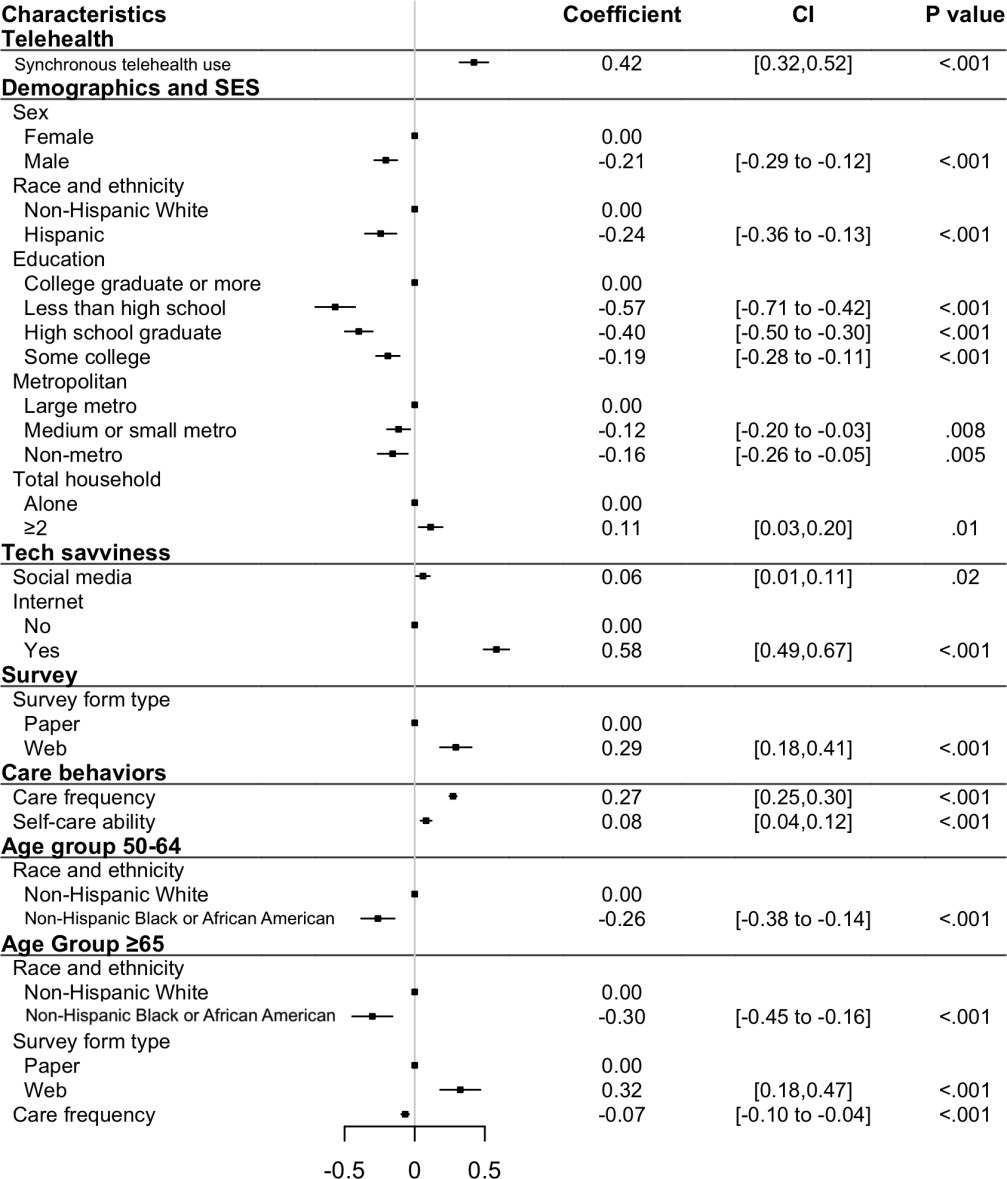
Significant variables identified from the patient portal model. OR: odds ratio; SES: socioeconomic status.

Using the selected variables, we constructed survey-weighted generalized linear models for the 3 outcomes. The adjusted *R*^2^ of the patient portal model was 0.31, while the pseudo-*R*^2^ values of the synchronous telehealth model and the Neither model were 0.15 and 0.22, respectively. Based on the acceptable range of 0.10-0.30 [21,22], all models exhibited a satisfactory goodness of fit. Figure 1 presents the unique and shared significant variables identified in the models, and Figures2^4^ detail the direction and strength of associations for each model.

**Figure 3.**
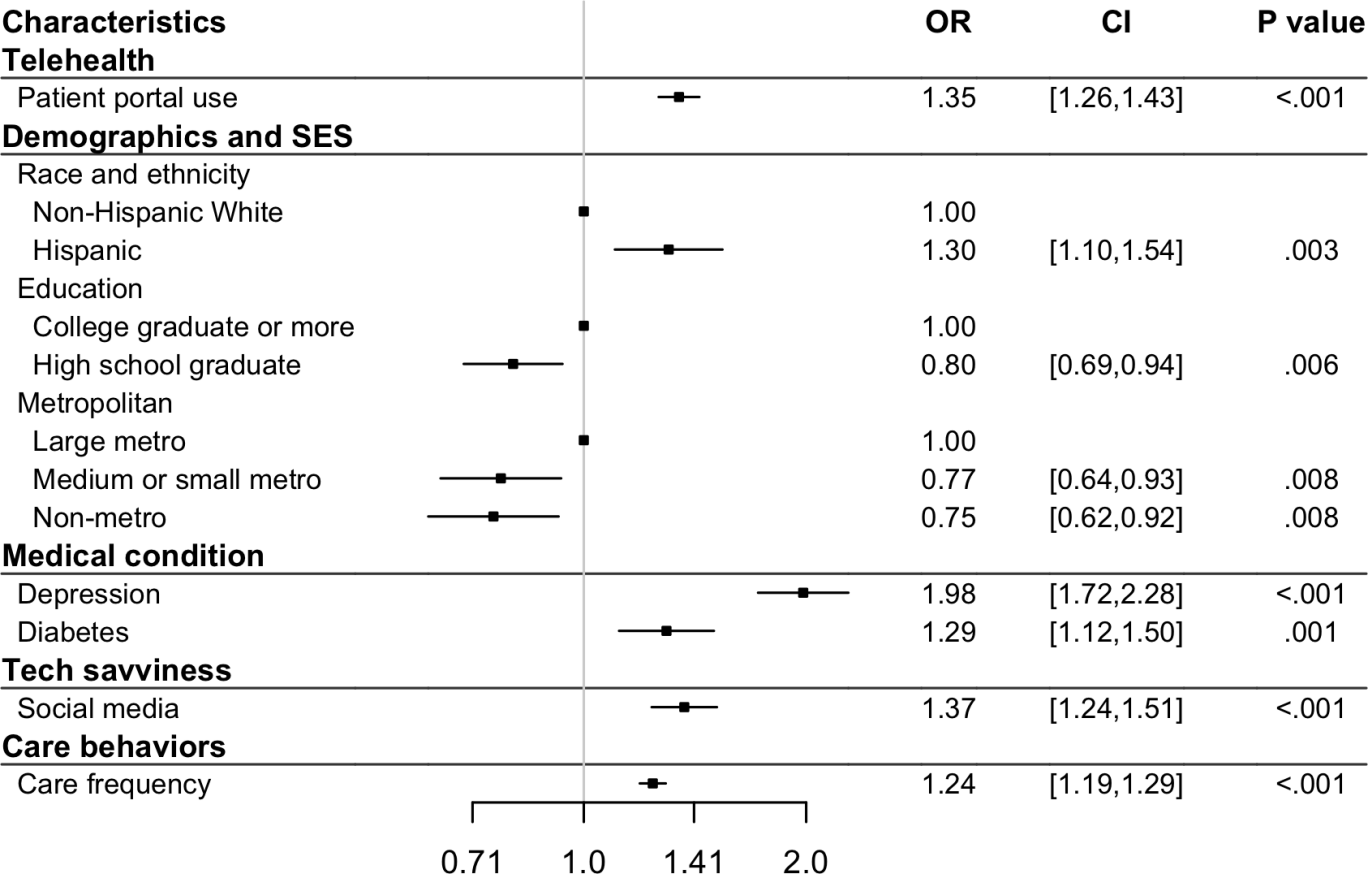
Significant variables identified from the synchronous telehealth model. OR: odds ratio; SES: socioeconomic status.

**Figure 4.**
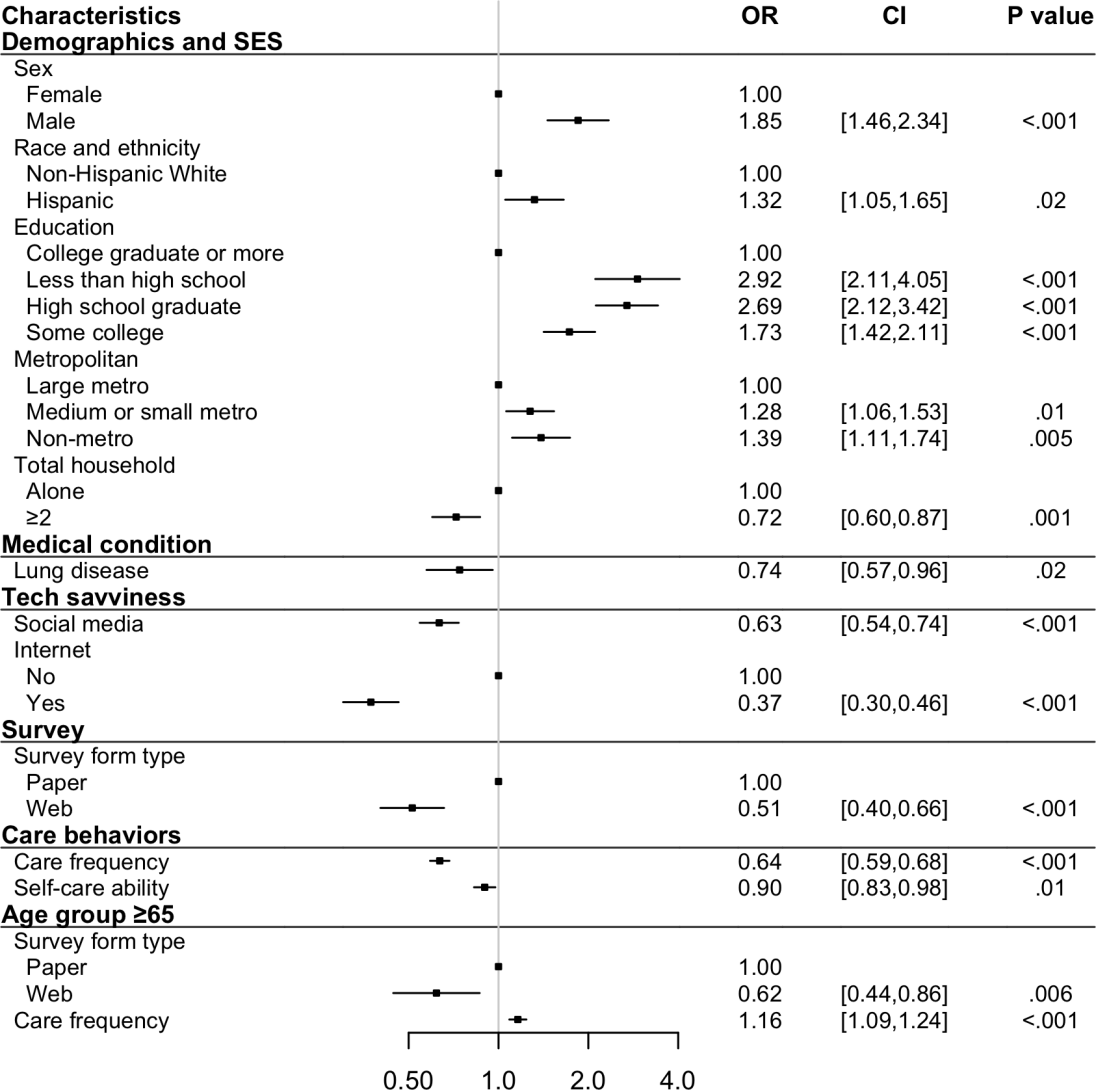
Significant variables identified from the neither model. OR: odds ratio; SES: socioeconomic status.

Use of one type of telehealth service was positively associated with use of the other modality (coefficient 0.42, 95% CI 0.32-0.53; *P*<.001 for patient portals; OR 1.35, 95% CI 1.26-1.43; *P*<.001 for synchronous). In terms of demographics and socioeconomic status, male patients were less likely to use patient portals than female (coefficient −0.21, 95% CI −0.29 to −0.12; *P*<.001), there was no significant difference in synchronous use between male and female patients (*P*=.10; see Table S6 in Multimedia Appendix 1). Additionally, Hispanic patients showed a contrasting pattern: they were more likely to use more synchronous telehealth (OR 1.30, 95% CI 1.10-1.54; *P*=.003) but less likely to use patient portals (coefficient −0.24, 95% CI −0.36 to −0.13; *P*<.001). Furthermore, middle-aged and older non-Hispanic Black or African American patients (aged 50 years and older) were less likely to use patient portals (coefficient −0.26, 95% CI −0.38 to −0.14; *P*<.001 for 50‐64 years; coefficient −0.30, 95% CI −0.45 to −0.16; *P*<.001 for ≥65 years).

Compared to patients in large metropolitan areas, those in medium or small metropolitan and nonmetropolitan areas were less likely to use either telehealth modality (patient portals: coefficient −0.12, 95% CI −0.20 to −0.03; *P*=.008 for medium or small metropolitan, coefficient −0.16, 95% CI −0.26 to −0.05; *P*=.005 for nonmetropolitan; synchronous: OR 0.77, 95% CI 0.64-0.93; *P*=.008 for medium or small metropolitan, OR 0.75, 95% CI 0.62-0.92; *P*=.008 for nonmetropolitan). Finally, among the demographic and socioeconomic variables, individuals living with other household members were more likely to use only patient portals (coefficient 0.11, 95% CI 0.03-0.20; *P*=.01). Those with higher educational attainment were more likely to use both services (patient portals: coefficient range [−0.57 to −0.19], 95% CI −0.71 to −0.11; *P*<.001 for all the other categories [less than high school, high school graduate, some college]; synchronous: OR 0.80, 95% CI 0.69-0.94; *P*=.006 for high school graduate; see Figures2  3).

Medical conditions played a role mainly for synchronous telehealth. Patients with depression were more likely to use synchronous telehealth services (OR 1.98, 95% CI 1.72-2.28; *P*<.001). Diabetes was associated with increased use of synchronous telehealth (OR 1.29, 95% CI 1.12-1.50; *P*=.001), and patients with lung disease were less likely to avoid telehealth altogether (OR 0.74, 95% CI 0.57-0.96; *P*=.02).

In the tech-savviness category, social media use was positively associated with telehealth use across both modalities (patient portals: coefficient 0.06, 95% CI 0.01-0.11; *P*=.02; synchronous: OR 1.37, 95% CI 1.24-1.51; *P*<.001). Moreover, those who responded using the internet were more likely to use patient portals (coefficient 0.58, 95% CI 0.49-0.67; *P*<.001). For the survey type, participants who completed web-based surveys were more likely to use patient portals than those who completed paper-based surveys (coefficient 0.29, 95% CI 0.18-0.41; *P*<.001), with this trend being even stronger among older adults (coefficient 0.32, 95% CI 0.18-0.47; *P*<.001).

Regarding care behaviors, patients who saw health care professionals more frequently were more likely to use telehealth (coefficient 0.27, 95% CI 0.25-0.30; *P*<.001 for patient portals; OR 1.24, 95% CI 1.19-1.29; *P*<.001 for synchronous). However, among older patients, this association was less pronounced for patient portals (coefficient −0.07, 95% CI −0.10 to −004; *P*<.001). Additionally, patients who reported being confident in managing their own health were more likely to use patient portals (coefficient 0.08, 95% CI 0.04-0.12; *P*<.001).

### Sensitivity Analyses

Table S5-S7 in Multimedia Appendix 1 exhibit the comparisons of the significant variables across models using the imputed dataset in the main text and 5 different imputations. Most significant variables identified in the main text models remained significant. However, due to the small number of samples in the age subgroups, the interaction terms sometimes became insignificant, non-Hispanic Black or African American in the patient portal models was identified as significant 3 out of 6 instances for both the 50‐64 years and ≥65 years age groups (see Table S6 in Multimedia Appendix 1).

As illustrated in Table S8-S10 in Multimedia Appendix 1, after excluding the form type variable, the effect of internet use became more pronounced in both the patient portal telehealth model (see Table S8 in Multimedia Appendix 1) and the Neither model (see Table S10 in Multimedia Appendix 1), where an OR closer to “0” indicates a much higher likelihood of using one or both telehealth options. For synchronous telehealth, the form type variable was not identified as significant in the original model, resulting in minimal change (see Table S9 in Multimedia Appendix 1).

Finally, the model treating patient portal use as a continuous variable largely shared significant predictors with the binary model, as shown in Table S11 in Multimedia Appendix 1. Each model, however, had a few unique significant variables: for the continuous model, nonmetro, social media use, and non-Hispanic Black or African American individuals aged 50‐64 years; for the binary model, high blood pressure and nonmetro status among those aged 65 years or older.

## Discussion

### Principal Findings

Using the harmonized HINTS Cycles 6 and 7 data, including national representative patient populations, this study offers a unique examination of both patient portal and synchronous telehealth use. While both services are delivered remotely and therefore share foundational similarities, their distinct functionalities lead to divergent patterns of use. By analyzing a range of factors, including demographics, socioeconomic status, and technological proficiency, we identified several common predictors associated with the use of both modalities, as well as unique factors specific to each. Furthermore, we discovered the variations across age groups, particularly among those aged 50‐64 years and those 65 years and older. These findings contribute to a deeper understanding of age-related differences in telehealth use and could offer insights for enhancing patient experience and promoting broader telehealth adoption.

Methodologically, our 2-step approach effectively addresses potential multicollinearity during variable selection and ensures nationally representative estimates. The bootstrapped variable selection process helps build a parsimonious set of variables while accommodating multiple interaction terms. With this reduced set of variables, the survey-weighted generalized linear models enable robust population-level statistical inference, providing both nationally representative estimates and measures of their variability.

### Unique Patterns of Telehealth Use Among Older Adults

While previous research has suggested that older adults are generally less likely to use telehealth [5,6], our models did not find age itself to be a significant predictor. Instead, our findings reveal that older adults exhibit unique usage patterns that differ from the general population in several ways; some are entirely new (eg, race-related differences), some are more pronounced (eg, survey form type), and others are weaker (eg, care frequency). These nuances underscore the importance of targeted investigations into telehealth use among older adults.

As an example of an emerging pattern, our data show non-Hispanic Black or African American patients overall used patient portals at rates similar to non-Hispanic White, which contradicts findings from prior research [25,26]. However, within this race group, individuals aged 50 years and older were less likely to use patient portals in some models trained on different imputed datasets. On average, responses shifted 0.26 points toward the “no-use” end of the scale for those aged 50‐64 years and 0.30 points for those aged 65 years and older. This suggests that the digital divide in patient portal services may not be solely age-based but could intersect with race, underscoring the possible need for multidimensional strategies to address disparities in the use.

In terms of stronger patterns in the old patient group (≥65 years), old patients who completed the web-based survey were more likely to use patient portals than the general population, with their reported usage increasing by an average of 0.32 points toward the “10 or more than 10 times” end of the scale. In the sensitivity analysis, completing a web-based survey likely reflects greater familiarity with technology. Taken together, these findings suggest that comfort with technology can significantly improve engagement with patient portal services. As such, targeted technology education initiatives for older adults could play a vital role in promoting patient portal adoption and improving health care accessibility for this population, as suggested in prior research [27].

Regarding weaker patterns observed in the old population (≥65 years), patients who frequently visited health care providers were more likely to use patient portals in the general population, but this association was weaker among older adults. For each one-point increase in visit frequency toward the “10 or more times” end of the scale, responses for patient portal use were, on average, 0.07 points lower. Patients who receive care more often are generally exposed to more educational opportunities about patient portals and have greater chances to use patient portals. However, older adults may prefer addressing all concerns during in-person visits rather than through patient portals.

### Key Differences in Drivers of Patient Portals and Synchronous Telehealth

The use of patient portals and synchronous telehealth shares some common drivers, such as residing in metropolitan areas and having higher levels of education. Particularly, patients who used one type of telehealth were more likely to use the other, consistent with previous studies suggesting that telehealth experience can enhance patient satisfaction and encourage further use [28,29]. However, important differences exist between the two. Patient portal use is largely shaped by patient characteristics, whereas synchronous telehealth depends primarily on the availability of real-time care options. Patients with diabetes and those with depression were more likely to use synchronous telehealth, with odds that were 98% higher and 29% higher, respectively, compared to those without these conditions. Additionally, those with lung disease were more likely to use either type or both, as their odds of not using any form of telehealth decreased by 26%. This finding likely reflects the nature of care required. For example, psychiatric care and diabetes care can involve synchronous consultations and telemonitoring [30,31]. Chronic obstructive pulmonary disease patients may involve a wide range of services, such as telerehabilitation, health education, telemonitoring, early detection of exacerbations, psychosocial support, and smoking cessation, delivered through both modalities [32,33]. These findings highlight the potential for synchronous telehealth patients who require long-term care and frequent visits.

On the other hand, the patient portal model included more variables related to tech-savviness (ie, internet use and completion of web-based surveys) in addition to social media use, which was commonly identified as significant for both modalities. Patients who used the internet scored, on average, 0.58 points higher toward the “10 or more than 10 times” end of the scale in patient portal usage, and those who completed the HINTS online scored 0.29 points higher. This may be due to the multiple functions available in patient portals, such as sending images and secure messages, which require a certain level of technological familiarity. In contrast, synchronous telehealth can be conducted not only via video but also through phone calls, and voice-only visits demand far fewer technical skills. Additionally, a one-unit increase in patients’ self-care ability toward the “very confident” end of the scale was associated with greater patient portal use, corresponding to an average increase of 0.08 points, but showed no association with synchronous telehealth use. This may be because patient portal features, such as viewing lab results or refilling prescriptions, are more directly aligned with self-management behaviors [34,35].

In terms of demographic and socioeconomic factors, prior research shows that male patients are generally less likely to use telehealth [36,37]. However, this study confirms that this pattern applies only to patient portal use, with an average decrease of 0.21 points toward the “no use” end of the scale, but not to synchronous telehealth. Additionally, patients living with household members were also more likely to use patient portals only. This pattern may be linked to the shared access features of patient portals [38]. Family members or care partners can log into the patient’s account to help manage their care, which may encourage patients to engage with the portal and participate more actively in their health care. Finally, age-specific effects were identified only for patient portal use. These findings suggest that promoting patient portal adoption should be particularly tailored to individual patient characteristics.

Interestingly, Hispanic patients showed contrasting usage patterns; they were more likely to use synchronous telehealth but less likely to use patient portals. Their odds of using synchronous telehealth increased by 30%, while their responses for patient portals were, on average, 0.24 points lower toward the “no use” end of the scale. This aligns with one side of the existing literature, which remains inconclusive [39,40]. One possible explanation is that while health care providers may offer services in Spanish, patient portal platforms often lack adequate translation features, creating barriers for patients with limited English proficiency [41]. To address this, multilingual support and culturally informed design are essential for effective telehealth delivery.

### Limitations

This study is based on observational, retrospective data from surveys not specifically designed for this research. As such, we were unable to explore in-depth questions, such as patient satisfaction with telehealth across both modalities and the underlying reasons for choosing patient portals and synchronous care. Furthermore, we applied survey weights to the final inference models to improve sample representativeness, but they could also be used for imputing missing values [42]. Additionally, despite the complex survey design approach, the generalizability of our findings remains limited. Survey weights adjust nonresponse based on observed characteristics, but unobserved factors may still influence participation. Residual confounding, such as broadband connectivity and state-level telehealth policies, both known to affect remote care use, was not directly measured. For example, counties with greater broadband availability have been shown to exhibit significantly higher telehealth use [43,44], and policy variations across states continue to impact access in rural areas [45]. Therefore, future research using multilevel designs or incorporating additional factors (eg, infrastructure and policy indicators) is warranted.

Another potential limitation is endogeneity between patient portal use and health care use frequency. Individuals who have more in-person health care service encounters may receive more provider recommendations to use patient portals, leading to higher portal adoption and engagement. Such unobserved factors may simultaneously drive greater portal use [46,47]. Future analyses could apply lagged models to test temporal ordering (eg, whether prior care use predicts later portal engagement) or use instrumental variable approaches, such as broadband access or provider-level promotion, to better account for endogeneity and unobserved confounding.

While harmonizing Cycles 6 and 7, we assumed no survey period effects. However, as shown in Table S12 in Multimedia Appendix 1, the use of patient portals and synchronous telehealth varied across cycles. In Cycle 7, participants reported more frequent use of patient portals and less frequent use of synchronous telehealth compared to Cycle 6 (all *P* values <.001 and chi-square tests). Future research should account for these period differences.

As shown in Table 2 and suggested in existing research [5,48], we recognize that disaggregating synchronous telehealth into video and phone visits could provide valuable insights. However, splitting synchronous encounters in our dataset resulted in small cell sizes within key age strata, leading to unstable estimates and reduced statistical power. Therefore, we retained a combined synchronous category in the primary analyses while noting potential modality differences as an important direction for future research with larger samples.

Finally, in the sensitivity analysis assessing potential bias from missing data imputation, race was sometimes identified as significant and at other times not. A possible explanation is the relatively higher missing rate for races (8.6%). Obtaining more complete observations would strengthen the robustness of our findings.

### Conclusions

This study identifies both shared and distinct factors significantly associated with patient portal and synchronous telehealth use, as well as age-specific use patterns. These insights contribute to a deeper understanding of how to effectively promote both modalities, particularly among aging populations. Notably, the increased use of patient portals among older adults who are more technologically adept suggests that ongoing digital education initiatives may enhance telehealth adoption in this demographic. Furthermore, targeted outreach to specific demographic groups among old patients, such as non-Hispanic Black or African American, may be necessary to address disparities in telehealth use. Collectively, these findings offer valuable guidance for optimizing telehealth implementation strategies and advancing equitable telehealth delivery.

## Supplementary material

10.2196/83730 Multimedia Appendix 1 Supplemental tables (Table S1 through S12) providing detailed results and additional analyses.

## Abbreviations

HINTS: Health Information National Trends Survey
LASSO: Least Absolute Shrinkage and Selection Operator
MICE: multivariate imputation by chained equation
OR: odds ratio
