# Supplementary material for: Identifying Factors Associated With Patient Portal and Synchronous Telehealth Use Across Age Groups in the Postpandemic Era: Retrospective Analysis of the Health Information National Trends Survey
Source: JMIR Aging. 2026 Mar 3;9:e83730. doi: 10.2196/83730 (PMC12977003; doi:10.2196/83730)
Supplement: Multimedia Appendix 1 [file aging-v9-e83730-s001.docx]

Table S1 Variable used in this study, their corresponding names and types in the database, and their missing rates.

| Variable name | Variable name in the HINTS Cycles 6 and 7 | Missing rate (%) |
| --- | --- | --- |
| Demographic and socio-economic status |  |  |
| Age | AgeGrpB | 3.7 |
| Sex | BirthGender for Cycle 6,  BirthSex for Cycle 7 | 5.2 |
| Race and ethnicity | RaceEthn5 | 8.6 |
| Total household | TotalHousehold | 5.3 |
| Education | EducA | 4.9 |
| Neighborhood | NCHSURCODE2013 | 0.0 |
| Medical condition |  |  |
| Depression | MedConditions_Depression | 2.1 |
| Diabetes | MedConditions_Diabetes | 2.2 |
| High blood pressure | MedConditions_HighBP | 2.0 |
| Heart condition | MedConditions_HeartCondition | 2.0 |
| Lung disease | MedConditions_LungDisease | 2.0 |
| Tech savviness |  |  |
| Social media interact | SocMed_Interacted | 1.0 |
| Use internet | UseInternet for Cycle 6,  FreqUseInternet for Cycle 7  (changed it to yes or no to match Cycle 6) | 0.2 |
| Survey |  |  |
| Form type | FormType for Cycle 6  FORMTYPE for Cycle 7 | 0.0 |
| Care behavior |  |  |
| Self-care ability | OwnAbilityTakeCareHealth | 1.5 |
| Care frequency | FreqGoProvider | 0.6 |
| Telehealth |  |  |
| Patient portal use | AccessOnlineRecord2 for Cycle 6,  AccessOnlineRecord3 for Cycle 7 | 0.0 |
| Synchronous telehealth use | ReceiveTelehealthCare | 0.0 |

Table S2 Variable selection for patient portals. Significant variables (P < .05) are in bold.

| Variables | Categories | mean | 95% lower | 95% upper | *P* | selection freq |
| --- | --- | --- | --- | --- | --- | --- |
| **Sync telehealth use** |  | 0.434 | 0.364 | 0.505 | 0.000 | 500 |
| Age | 50-64 | 0.038 | -0.094 | 0.170 | 0.572 | 213 |
|  | ≥65 | -0.008 | -0.077 | 0.061 | 0.826 | 80 |
| **Sex** | **Male** | -0.208 | -0.266 | -0.151 | 0.000 | 500 |
| **Race and ethnicity** | Non-Hispanic Black or African American | -0.078 | -0.176 | 0.021 | 0.121 | 471 |
|  | **Hispanic** | -0.223 | -0.301 | -0.144 | 0.000 | 500 |
|  | Non-Hispanic Asian | -0.006 | -0.097 | 0.086 | 0.900 | 362 |
|  | Non-Hispanic Other | -0.049 | -0.177 | 0.080 | 0.458 | 415 |
| **Total household** | **≥2** | 0.139 | 0.074 | 0.205 | 0.000 | 500 |
| **Education** | **Less than high school** | -0.574 | -0.704 | -0.444 | 0.000 | 500 |
|  | **High school graduate** | -0.431 | -0.511 | -0.351 | 0.000 | 500 |
|  | **Some college** | -0.251 | -0.324 | -0.179 | 0.000 | 500 |
| **Neighborhood** | Large fringe metro | 0.034 | -0.030 | 0.099 | 0.298 | 426 |
|  | **Medium or small metro** | -0.108 | -0.177 | -0.039 | 0.002 | 500 |
|  | **Non-metro** | -0.104 | -0.201 | -0.007 | 0.036 | 484 |
| **Depression** | **Yes** | 0.065 | 0.001 | 0.129 | 0.047 | 482 |
| Diabetes | Yes | 0.030 | -0.040 | 0.100 | 0.403 | 397 |
| High blood pressure | Yes | 0.052 | -0.012 | 0.117 | 0.112 | 481 |
| Heart condition | Yes | -0.039 | -0.162 | 0.084 | 0.533 | 325 |
| Lung disease | Yes | 0.068 | -0.024 | 0.160 | 0.149 | 467 |
| **Social media interact** |  | 0.084 | 0.048 | 0.119 | 0.000 | 500 |
| **Use internet** | **Yes** | 0.519 | 0.411 | 0.626 | 0.000 | 500 |
| **Form type** | **web** | 0.339 | 0.257 | 0.421 | 0.000 | 500 |
| **Self-care ability** |  | 0.061 | 0.036 | 0.086 | 0.000 | 500 |
| **Care frequency** | **None** | 0.265 | 0.245 | 0.285 | 0.000 | 500 |
| *Age 50-64* |  |  |  |  |  |  |
| Synchronous telehealth use |  | -0.088 | -0.197 | 0.021 | 0.113 | 468 |
| Sex | Male | 0.017 | -0.061 | 0.095 | 0.662 | 354 |
| **Race and ethnicity** | **Non-Hispanic Black or African American** | -0.181 | -0.327 | -0.036 | 0.015 | 496 |
|  | **Hispanic** | -0.139 | -0.276 | -0.002 | 0.046 | 488 |
|  | Non-Hispanic Asian | -0.085 | -0.281 | 0.112 | 0.398 | 417 |
|  | Non-Hispanic Other | -0.107 | -0.334 | 0.120 | 0.354 | 426 |
| Total household | ≥2 | 0.030 | -0.055 | 0.115 | 0.491 | 359 |
| Education | Less than high school | -0.189 | -0.390 | 0.012 | 0.066 | 485 |
|  | High school graduate | -0.053 | -0.170 | 0.063 | 0.369 | 406 |
|  | Some college | 0.040 | -0.056 | 0.135 | 0.415 | 395 |
| Neighborhood | Large fringe metro | 0.005 | -0.085 | 0.096 | 0.911 | 338 |
|  | Medium or small metro | 0.029 | -0.066 | 0.123 | 0.549 | 362 |
|  | Non-metro | -0.036 | -0.163 | 0.092 | 0.583 | 375 |
| Depression |  | 0.013 | -0.085 | 0.111 | 0.793 | 364 |
| Diabetes |  | 0.035 | -0.062 | 0.131 | 0.479 | 379 |
| High blood pressure |  | 0.000 | -0.081 | 0.080 | 0.994 | 321 |
| Heart condition |  | 0.042 | -0.139 | 0.222 | 0.652 | 381 |
| Lung disease |  | -0.052 | -0.190 | 0.085 | 0.454 | 408 |
| Social media interact |  | 0.026 | -0.030 | 0.082 | 0.359 | 387 |
| Use internet | Yes | 0.034 | -0.069 | 0.138 | 0.516 | 259 |
| Form type | Web | 0.108 | -0.006 | 0.221 | 0.063 | 489 |
| Self-care ability |  | -0.006 | -0.038 | 0.026 | 0.697 | 226 |
| Care frequency |  | -0.013 | -0.040 | 0.014 | 0.337 | 336 |
| *Age ≥65* |  |  |  |  |  |  |
| **Synchronous telehealth use** |  | -0.124 | -0.228 | -0.021 | 0.019 | 494 |
| Sex | Male | 0.048 | -0.034 | 0.130 | 0.254 | 425 |
| **Race and ethnicity** | **Non-Hispanic Black or African American** | -0.209 | -0.349 | -0.069 | 0.004 | 499 |
|  | Hispanic | -0.110 | -0.233 | 0.013 | 0.079 | 476 |
|  | Non-Hispanic Asian | -0.222 | -0.451 | 0.008 | 0.058 | 492 |
|  | Non-Hispanic Other | -0.183 | -0.426 | 0.060 | 0.139 | 470 |
| Total household | ≥2 | 0.025 | -0.053 | 0.104 | 0.528 | 367 |
| Education | Less than high school | 0.050 | -0.104 | 0.204 | 0.522 | 352 |
|  | High school graduate | -0.001 | -0.089 | 0.088 | 0.991 | 328 |
|  | Some college | 0.023 | -0.070 | 0.117 | 0.626 | 351 |
| Neighborhood | Large fringe metro | 0.078 | -0.028 | 0.184 | 0.147 | 463 |
|  | Medium or small metro | 0.030 | -0.060 | 0.120 | 0.508 | 358 |
|  | Non-metro | -0.070 | -0.194 | 0.054 | 0.267 | 424 |
| Depression |  | -0.015 | -0.111 | 0.081 | 0.762 | 382 |
| Diabetes |  | 0.000 | -0.085 | 0.086 | 0.991 | 312 |
| High blood pressure |  | -0.006 | -0.077 | 0.065 | 0.876 | 290 |
| Heart condition |  | 0.045 | -0.099 | 0.189 | 0.538 | 352 |
| Lung disease |  | 0.023 | -0.089 | 0.135 | 0.684 | 353 |
| Social media interact |  | 0.030 | -0.028 | 0.087 | 0.314 | 385 |
| Use internet | Yes | 0.060 | -0.057 | 0.178 | 0.315 | 381 |
| **Form type** | **Web** | 0.279 | 0.169 | 0.389 | 0.000 | 500 |
| Self-care ability |  | -0.003 | -0.026 | 0.021 | 0.820 | 235 |
| **Care frequency** |  | -0.067 | -0.098 | -0.037 | 0.000 | 500 |

Table S3 Variable selection for synchronous telehealth. Significant variables (P < .05) are in bold.

| Variables | Categories | mean | 95% lower | 95% upper | *P* | selection freq |
| --- | --- | --- | --- | --- | --- | --- |
| **Patient portal use** |  | 0.304 | 0.253 | 0.355 | 0.000 | 500 |
| Age | 50-64 | -0.127 | -0.506 | 0.253 | 0.513 | 248 |
|  | ≥65 | 0.059 | -0.191 | 0.309 | 0.644 | 147 |
| **Sex** | **Male** | -0.189 | -0.320 | -0.059 | 0.004 | 500 |
| Race and ethnicity | Non-Hispanic Black or African American | 0.035 | -0.099 | 0.169 | 0.609 | 350 |
|  | **Hispanic** | 0.267 | 0.121 | 0.413 | 0.000 | 500 |
|  | Non-Hispanic Asian | 0.128 | -0.077 | 0.332 | 0.221 | 441 |
|  | Non-Hispanic Other | 0.025 | -0.183 | 0.232 | 0.814 | 346 |
| Total household | ≥2 | 0.047 | -0.056 | 0.149 | 0.371 | 407 |
| Education | **Less than high school** | -0.278 | -0.531 | -0.024 | 0.032 | 492 |
|  | **High school graduate** | -0.252 | -0.410 | -0.093 | 0.002 | 499 |
|  | Some college | -0.098 | -0.218 | 0.022 | 0.108 | 469 |
| Neighborhood | Large fringe metro | 0.015 | -0.101 | 0.131 | 0.803 | 340 |
|  | **Medium small metro** | -0.266 | -0.401 | -0.130 | 0.000 | 500 |
|  | **Non-metro** | -0.305 | -0.531 | -0.079 | 0.008 | 500 |
| Depression | **Yes** | 0.607 | 0.470 | 0.743 | 0.000 | 500 |
| Diabetes | **Yes** | 0.248 | 0.089 | 0.406 | 0.002 | 497 |
| High blood pressure | Yes | 0.014 | -0.081 | 0.109 | 0.766 | 320 |
| Heart condition | Yes | 0.106 | -0.142 | 0.355 | 0.400 | 360 |
| Lung disease | Yes | 0.169 | -0.009 | 0.347 | 0.062 | 474 |
| **Social media internet** |  | 0.273 | 0.214 | 0.332 | 0.000 | 500 |
| Use internet | Yes | -0.092 | -0.257 | 0.072 | 0.271 | 427 |
| Form type | web | 0.083 | -0.043 | 0.208 | 0.196 | 449 |
| Self-care ability |  | -0.004 | -0.045 | 0.036 | 0.837 | 374 |
| **Care frequency** | **None** | 0.187 | 0.156 | 0.219 | 0.000 | 500 |
| *Age 50-64* |  |  |  |  |  |  |
| Patient portal |  | -0.068 | -0.142 | 0.006 | 0.072 | 481 |
| Sex | Male | 0.131 | -0.062 | 0.324 | 0.182 | 439 |
| Race and ethnicity | Non-Hispanic Black or African American | 0.140 | -0.079 | 0.359 | 0.211 | 441 |
|  | Hispanic | 0.197 | -0.042 | 0.435 | 0.106 | 476 |
|  | Non-Hispanic Asian | -0.083 | -0.470 | 0.304 | 0.674 | 407 |
|  | Non-Hispanic Other | 0.421 | -0.044 | 0.886 | 0.076 | 478 |
| Total household | ≥2 | 0.003 | -0.133 | 0.139 | 0.968 | 297 |
| Education | Less than high school | 0.025 | -0.288 | 0.339 | 0.874 | 345 |
|  | High school graduate | -0.006 | -0.202 | 0.191 | 0.954 | 337 |
|  | Some college | -0.025 | -0.195 | 0.144 | 0.768 | 368 |
| Neighborhood | Large fringe metro | -0.064 | -0.241 | 0.113 | 0.477 | 369 |
|  | Medium or small metro | -0.072 | -0.268 | 0.125 | 0.474 | 391 |
|  | Non-metro | -0.244 | -0.568 | 0.079 | 0.139 | 471 |
| Depression |  | 0.183 | -0.027 | 0.393 | 0.087 | 482 |
| Diabetes |  | -0.156 | -0.381 | 0.069 | 0.174 | 431 |
| High blood pressure |  | -0.022 | -0.164 | 0.121 | 0.766 | 332 |
| Heart condition |  | -0.025 | -0.316 | 0.267 | 0.868 | 347 |
| Lung disease |  | 0.174 | -0.085 | 0.433 | 0.188 | 452 |
| Social media interact |  | -0.014 | -0.096 | 0.068 | 0.743 | 307 |
| Use internet | Yes | 0.012 | -0.175 | 0.199 | 0.897 | 178 |
| Form type | Web | 0.009 | -0.138 | 0.156 | 0.904 | 274 |
| Self-care ability |  | 0.028 | -0.044 | 0.100 | 0.453 | 295 |
| Care frequency |  | 0.011 | -0.028 | 0.051 | 0.565 | 282 |
| *Age ≥65* |  |  |  |  |  |  |
| **Patient portal use** |  | -0.096 | -0.167 | -0.025 | 0.008 | 499 |
| Sex | Male | 0.151 | -0.030 | 0.332 | 0.102 | 467 |
| Race and ethnicity | Non-Hispanic Black or African American | 0.180 | -0.047 | 0.406 | 0.120 | 463 |
|  | Hispanic | 0.158 | -0.068 | 0.385 | 0.169 | 464 |
|  | Non-Hispanic Asian | -0.067 | -0.444 | 0.310 | 0.728 | 400 |
|  | Non-Hispanic Other | 0.130 | -0.253 | 0.514 | 0.505 | 417 |
| Total household | ≥2 | 0.087 | -0.067 | 0.242 | 0.268 | 415 |
| Education | Less than high school | 0.057 | -0.250 | 0.364 | 0.716 | 339 |
|  | High school graduate | -0.048 | -0.243 | 0.148 | 0.632 | 351 |
|  | Some college | -0.056 | -0.208 | 0.095 | 0.465 | 371 |
| Neighborhood | Large fringe metro | -0.160 | -0.358 | 0.038 | 0.113 | 475 |
|  | Medium or small metro | 0.035 | -0.120 | 0.190 | 0.657 | 343 |
|  | **Non-metro** | -0.295 | -0.588 | -0.003 | 0.048 | 486 |
| Depression |  | -0.161 | -0.367 | 0.045 | 0.125 | 466 |
| Diabetes |  | 0.060 | -0.127 | 0.247 | 0.528 | 368 |
| High blood pressure |  | 0.072 | -0.080 | 0.225 | 0.352 | 381 |
| Heart condition |  | -0.126 | -0.409 | 0.156 | 0.381 | 376 |
| Lung disease |  | 0.097 | -0.129 | 0.323 | 0.397 | 385 |
| Social media interact |  | -0.003 | -0.087 | 0.080 | 0.939 | 297 |
| Use internet | Yes | -0.016 | -0.167 | 0.134 | 0.831 | 206 |
| Form type | Web | 0.109 | -0.059 | 0.277 | 0.202 | 425 |
| Self-care ability |  | -0.024 | -0.081 | 0.034 | 0.417 | 343 |
| Care frequency |  | -0.021 | -0.062 | 0.019 | 0.300 | 394 |

Table S4 Variable selection for the Neither outcome. Significant variables (P < .05) are in bold.

| Variables | Categories | mean | 95% lower | 95% upper | *P* | selection freq |
| --- | --- | --- | --- | --- | --- | --- |
| Age | 50-64 | 0.053 | -0.274 | 0.380 | 0.750 | 176 |
|  | ≥65 | -0.060 | -0.367 | 0.246 | 0.699 | 190 |
| **Sex** | **Male** | 0.526 | 0.390 | 0.662 | 0.000 | 500 |
| Race and ethnicity | Non-Hispanic Black or African American | 0.122 | -0.077 | 0.320 | 0.230 | 443 |
|  | **Hispanic** | 0.423 | 0.241 | 0.605 | 0.000 | 500 |
|  | Non-Hispanic Asian | -0.004 | -0.229 | 0.220 | 0.969 | 411 |
|  | Non-Hispanic Other | 0.088 | -0.212 | 0.389 | 0.564 | 409 |
| **Total household** | **≥2** | -0.320 | -0.493 | -0.147 | 0.000 | 500 |
| **Education** | **Less than high school** | 1.246 | 0.914 | 1.578 | 0.000 | 500 |
|  | **High school graduate** | 0.947 | 0.741 | 1.154 | 0.000 | 500 |
|  | **Some college** | 0.621 | 0.425 | 0.818 | 0.000 | 500 |
| **Neighborhood** | **LargeFringeMetro** | -0.238 | -0.428 | -0.047 | 0.015 | 497 |
|  | **MediumSmallMetro** | 0.299 | 0.114 | 0.484 | 0.002 | 500 |
|  | **NonMetro** | 0.451 | 0.228 | 0.674 | 0.000 | 500 |
| **Depression** | **Yes** | -0.215 | -0.385 | -0.045 | 0.013 | 495 |
| Diabetes | Yes | -0.235 | -0.480 | 0.009 | 0.059 | 479 |
| High blood pressure | Yes | -0.131 | -0.287 | 0.024 | 0.098 | 481 |
| Heart condition | Yes | -0.332 | -0.882 | 0.217 | 0.235 | 420 |
| **Lung disease** | **Yes** | -0.255 | -0.490 | -0.019 | 0.034 | 485 |
| **Social media interact** |  | -0.426 | -0.524 | -0.328 | 0.000 | 500 |
| **Use internet** | **Yes** | -0.730 | -1.015 | -0.446 | 0.000 | 500 |
| **Form type** | **web** | -0.696 | -0.885 | -0.506 | 0.000 | 500 |
| **Self-care ability** |  | -0.110 | -0.176 | -0.043 | 0.001 | 500 |
| **Care frequency** | **None** | -0.479 | -0.537 | -0.421 | 0.000 | 500 |
| *Age 50-64* |  |  |  |  |  |  |
| Sex | Male | -0.051 | -0.240 | 0.138 | 0.598 | 388 |
| Race and ethnicity | Non-Hispanic Black or African American | 0.212 | -0.094 | 0.518 | 0.173 | 471 |
|  | Hispanic | -0.151 | -0.438 | 0.136 | 0.301 | 450 |
|  | Non-Hispanic Asian | 0.039 | -0.371 | 0.449 | 0.853 | 406 |
|  | Non-Hispanic Other | -0.494 | -1.115 | 0.127 | 0.119 | 474 |
| Total household | ≥2 | -0.146 | -0.372 | 0.079 | 0.203 | 459 |
| Education | Less than high school | -0.008 | -0.281 | 0.266 | 0.956 | 409 |
|  | High school graduate | 0.042 | -0.394 | 0.478 | 0.849 | 413 |
|  | Some college | -0.267 | -0.551 | 0.017 | 0.065 | 478 |
| Neighborhood | Large fringe metro | 0.157 | -0.113 | 0.428 | 0.253 | 444 |
|  | Medium or small metro | -0.052 | -0.309 | 0.206 | 0.693 | 406 |
|  | Non-metro | 0.011 | -0.277 | 0.299 | 0.938 | 394 |
| Depression |  | -0.196 | -0.466 | 0.074 | 0.155 | 465 |
| Diabetes |  | 0.269 | -0.044 | 0.583 | 0.092 | 478 |
| High blood pressure |  | -0.025 | -0.230 | 0.180 | 0.811 | 375 |
| Heart condition |  | 0.353 | -0.309 | 1.015 | 0.295 | 427 |
| Lung disease |  | -0.075 | -0.435 | 0.285 | 0.682 | 395 |
| Social media interact |  | 0.048 | -0.100 | 0.195 | 0.527 | 345 |
| Use internet | Yes | -0.108 | -0.426 | 0.210 | 0.505 | 325 |
| Form type | Web | -0.109 | -0.337 | 0.119 | 0.349 | 397 |
| Self-care ability |  | -0.021 | -0.096 | 0.055 | 0.592 | 311 |
| **Care frequency** |  | 0.098 | 0.019 | 0.178 | 0.016 | 496 |
| *Age ≥65* |  |  |  |  |  |  |
| **Sex** | **Male** | -0.270 | -0.473 | -0.067 | 0.009 | 495 |
| Race and ethnicity | Non-Hispanic Black or African American | 0.064 | -0.202 | 0.330 | 0.636 | 415 |
|  | Hispanic | -0.171 | -0.432 | 0.091 | 0.200 | 467 |
|  | Non-Hispanic Asian | 0.218 | -0.230 | 0.666 | 0.339 | 454 |
|  | Non-Hispanic Other | 0.222 | -0.258 | 0.702 | 0.364 | 461 |
| Total household | ≥2 | -0.027 | -0.225 | 0.170 | 0.788 | 355 |
| Education | Less than high school | 0.037 | -0.107 | 0.181 | 0.615 | 349 |
|  | High school graduate | -0.420 | -0.872 | 0.031 | 0.068 | 481 |
|  | Some college | -0.093 | -0.351 | 0.164 | 0.477 | 405 |
| Neighborhood | Large fringe metro | -0.219 | -0.476 | 0.039 | 0.096 | 473 |
|  | Medium or small metro | -0.011 | -0.251 | 0.229 | 0.927 | 396 |
|  | Non-metro | -0.098 | -0.321 | 0.125 | 0.388 | 428 |
| Depression |  | 0.065 | -0.180 | 0.310 | 0.602 | 396 |
| Diabetes |  | 0.028 | -0.188 | 0.243 | 0.799 | 410 |
| High blood pressure |  | -0.013 | -0.276 | 0.250 | 0.923 | 357 |
| Heart condition |  | -0.010 | -0.193 | 0.172 | 0.911 | 343 |
| Lung Disease |  | 0.437 | -0.134 | 1.008 | 0.133 | 482 |
| Social Media Interact |  | -0.069 | -0.339 | 0.202 | 0.618 | 382 |
| Use Internet | Yes | -0.202 | -0.517 | 0.113 | 0.209 | 448 |
| **Form Type** | **Web** | -0.382 | -0.624 | -0.140 | 0.002 | 499 |
| Own Ability Take Care Health |  | 0.040 | -0.042 | 0.121 | 0.342 | 361 |
| **Frequent Go Provider** |  | 0.221 | 0.149 | 0.294 | 0.000 | 500 |

Table S5 Sensitivity analysis using different imputed datasets for aysnchronous telehealth.

|  | Original | | Imputed 1 | | Imputed 2 | | Imputed 3 | | Imputed 4 | | Imputed 5 | |
| --- | --- | --- | --- | --- | --- | --- | --- | --- | --- | --- | --- | --- |
|  | Coef | *P* | Coef | *P* | Coef | *P* | Coef | *P* | Coef | *P* | Coef | *P* |
| Intercept | -0.44 | <.001 | -0.46 | <.001 | -0.42 | <.001 | -0.42 | <.001 | -0.40 | <.001 | -0.43 | <.001 |
| Telehealth |  |  |  |  |  |  |  |  |  |  |  |  |
| Sync telehealth | 0.42 | <.001 | 0.41 | <.001 | 0.44 | <.001 | 0.42 | <.001 | 0.43 | <.001 | 0.41 | <.001 |
| Demo and SES |  |  |  |  |  |  |  |  |  |  |  |  |
| Male | -0.21 | <.001 | -0.22 | <.001 | -0.21 | <.001 | -0.20 | <.001 | -0.22 | <.001 | -0.23 | <.001 |
| Non-Hispanic Black or African American |  |  | -0.24 | <.001 |  |  | -0.14 | .11 |  |  | -0.19 | .03 |
| Hispanic | -0.24 | <.001 | -0.24 | <.001 | -0.24 | <.001 | -0.27 | <.001 | -0.26 | <.001 | -0.25 | <.001 |
| High school graduate | -0.40 | <.001 | -0.40 | <.001 | -0.42 | <.001 | -0.39 | <.001 | -0.43 | <.001 | -0.38 | <.001 |
| Less than high school | -0.57 | <.001 | -0.57 | <.001 | -0.59 | <.001 | -0.53 | <.001 | -0.54 | <.001 | -0.53 | <.001 |
| Some college | -0.19 | <.001 | -0.19 | <.001 | -0.18 | <.001 | -0.17 | <.001 | -0.19 | <.001 | -0.15 | <.001 |
| Medium or small metro | -0.12 | .01 | -0.12 | <.001 | -0.11 | .01 | -0.12 | .01 | -0.12 | .01 | -0.13 | <.001 |
| Non-metro | -0.16 | .01 | -0.18 | <.001 | -0.16 | <.001 | -0.17 | <.001 | -0.17 | <.001 | -0.18 | <.001 |
| Total household ≥2 | 0.11 | .01 | 0.11 | .01 | 0.13 | <.001 | 0.12 | <.001 | 0.13 | <.001 | 0.12 | .01 |
| Medical Condition |  |  |  |  |  |  |  |  |  |  |  |  |
| Depression | 0.01 | .84 |  |  |  |  | 0.02 | .63 | 0.04 | .44 | 0.03 | .57 |
| High blood pressure |  |  | 0.12 | .01 |  |  |  |  |  |  |  |  |
| Tech Savviness |  |  |  |  |  |  |  |  |  |  |  |  |
| Social media | 0.06 | .02 | 0.06 | .01 | 0.06 | .02 | 0.06 | .03 | 0.06 | .03 | 0.05 | .04 |
| Internet use | 0.58 | <.001 | 0.59 | <.001 | 0.58 | <.001 | 0.58 | <.001 | 0.58 | <.001 | 0.60 | <.001 |
| Survey |  |  |  |  |  |  |  |  |  |  |  |  |
| Survey form type | 0.29 | <.001 | 0.31 | <.001 | 0.28 | <.001 | 0.30 | <.001 | 0.29 | <.001 | 0.30 | <.001 |
| Care Behavior |  |  |  |  |  |  |  |  |  |  |  |  |
| Self-care ability | 0.08 | <.001 | 0.09 | <.001 | 0.08 | <.001 | 0.08 | <.001 | 0.08 | <.001 | 0.08 | <.001 |
| Care frequency | 0.27 | <.001 | 0.27 | <.001 | 0.27 | <.001 | 0.27 | <.001 | 0.27 | <.001 | 0.27 | <.001 |
| Age group 50-64 |  |  |  |  |  |  |  |  |  |  |  |  |
| Sync telehealth | -0.02 | .82 |  |  | -0.04 | .60 | -0.03 | .71 | -0.04 | .64 |  |  |
| Non-Hispanic Black or African American | -0.26 | <.001 |  |  | -0.25 | <.001 | -0.12 | .20 | -0.27 | <.001 | -0.11 | .24 |
| Hispanic | 0.07 | .53 |  |  |  |  |  |  | 0.08 | .49 |  |  |
| Age group ≥65 |  |  |  |  |  |  |  |  |  |  |  |  |
| Non-Hispanic Black or African American | -0.30 | <.001 | -0.10 | .29 | -0.31 | <.001 | -0.18 | .11 | -0.30 | <.001 | -0.11 | .30 |
| Survey form type | 0.32 | <.001 | 0.30 | <.001 | 0.33 | <.001 | 0.29 | <.001 | 0.32 | <.001 | 0.31 | <.001 |
| Care frequency | -0.07 | <.001 | -0.08 | <.001 | -0.07 | <.001 | -0.07 | <.001 | -0.06 | <.001 | -0.07 | <.001 |

Table S6 Sensitivity analysis using different imputed datasets for synchronous telehealth.

|  | Original | | Imputed 1 | | Imputed 2 | | Imputed 3 | | Imputed 4 | | Imputed 5 | |
| --- | --- | --- | --- | --- | --- | --- | --- | --- | --- | --- | --- | --- |
|  | OR | *P* | OR | *P* | OR | *P* | OR | *P* | OR | *P* | OR | *P* |
| (Intercept) | 0.10 | <.001 | 0.10 | <.001 | 0.09 | <.001 | 0.09 | <.001 | 0.09 | <.001 | 0.10 | <.001 |
| Telehealth |  |  |  |  |  |  |  |  |  |  |  |  |
| Patient Portal | 1.35 | <.001 | 1.35 | <.001 | 1.36 | <.001 | 1.36 | <.001 | 1.35 | <.001 | 1.35 | <.001 |
| Demo and SES |  |  |  |  |  |  |  |  |  |  |  |  |
| Male | 0.90 | .10 | 0.91 | .14 | 0.92 | .18 | 0.90 | .10 | 0.92 | .17 | 0.90 | .12 |
| Hispanic | 1.30 | .003 | 1.30 | <.001 | 1.28 | .01 | 1.28 | <.001 | 1.29 | <.001 | 1.24 | .01 |
| High school graduate | 0.80 | .01 | 0.80 | .01 | 0.89 | .27 | 0.81 | .01 | 0.88 | .19 | 0.82 | .01 |
| Less than high school | 0.78 | .08 | 0.77 | .07 |  |  |  |  |  |  |  |  |
| Medium or small metro | 0.77 | .01 | 0.77 | .01 | 0.77 | .01 | 0.77 | .01 | 0.77 | .01 | 0.77 | .01 |
| Non-metro | 0.75 | .01 | 0.75 | .01 | 0.75 | .01 | 0.70 | <.001 | 0.75 | .01 | 0.70 | <.001 |
| Medical Condition |  |  |  |  |  |  |  |  |  |  |  |  |
| Depression | 1.98 | <.001 | 1.92 | <.001 | 1.96 | <.001 | 1.86 | <.001 | 1.97 | <.001 | 1.99 | <.001 |
| Diabetes | 1.29 | .001 | 1.27 | <.001 | 1.26 | <.001 | 1.24 | <.001 | 1.25 | <.001 | 1.25 | <.001 |
| Lung disease |  |  | 1.37 | <.001 |  |  | 1.35 | <.001 |  |  |  |  |
| Tech Savviness |  |  |  |  |  |  |  |  |  |  |  |  |
| Social media | 1.37 | <.001 | 1.37 | <.001 | 1.37 | <.001 | 1.37 | <.001 | 1.36 | <.001 | 1.37 | <.001 |
| Care behavior |  |  |  |  |  |  |  |  |  |  |  |  |
| Care frequency | 1.24 | <.001 | 1.23 | <.001 | 1.23 | <.001 | 1.24 | <.001 | 1.25 | <.001 | 1.24 | <.001 |
| Age group ≥65 |  |  |  |  |  |  |  |  |  |  |  |  |
| Patient Portal | 0.96 | .21 | 0.95 | .17 | 0.96 | 0.27 | 0.94 | .09 | 0.95 | .17 | 0.95 | .12 |
| Non-metro | 0.78 | .12 | 0.77 | .11 | 0.75 | 0.08 |  |  | 0.77 | .11 |  |  |
| Depression |  |  |  |  |  |  | 1.01 | .97 | 0.99 | .92 | 1.00 | .98 |

Table S7 Sensitivity analysis using different imputed datasets for the Neither outcome.

|  | Original | | Imputed 1 | | Imputed 2 | | Imputed 3 | | Imputed 4 | | Imputed 5 | |
| --- | --- | --- | --- | --- | --- | --- | --- | --- | --- | --- | --- | --- |
|  | OR | *P* | OR | *P* | OR | *P* | OR | *P* | OR | *P* | OR | *P* |
| (Intercept) | 8.21 | <.001 | 9.77 | <.001 | 8.61 | <.001 | 8.78 | <.001 | 9.40 | <.001 | 7.78 | <.001 |
| Demo and SES |  |  |  |  |  |  |  |  |  |  |  |  |
| Male | 1.85 | <.001 | 1.87 | <.001 | 1.79 | <.001 | 1.92 | <.001 | 1.88 | <.001 | 1.90 | <.001 |
| Hispanic | 1.32 | .02 | 1.31 | .02 | 1.33 | .02 | 1.41 | <.001 | 1.34 | .01 | 1.32 | .02 |
| Total household ≥2 | 0.72 | <.001 | 0.72 | <.001 | 0.69 | <.001 | 0.76 | .01 | 0.69 | <.001 | 0.74 | <.001 |
| High school graduate | 2.69 | <.001 | 2.74 | <.001 | 2.72 | <.001 | 2.94 | <.001 | 2.98 | <.001 | 2.81 | <.001 |
| Less than high school | 2.92 | <.001 | 3.42 | <.001 | 3.34 | <.001 | 3.44 | <.001 | 3.35 | <.001 | 3.32 | <.001 |
| Some college | 1.73 | <.001 | 1.77 | <.001 | 1.98 | <.001 | 1.86 | <.001 | 1.97 | <.001 | 2.04 | <.001 |
| Large fringe metro | 0.78 | .05 | 0.77 | .04 | 0.77 | .04 | 0.77 | .04 | 0.76 | .03 | 0.77 | .05 |
| Medium or small metro | 1.28 | .01 | 1.27 | .01 | 1.26 | .02 | 1.29 | .01 | 1.26 | .02 | 1.27 | .01 |
| Non-metro | 1.39 | .01 | 1.40 | <.001 | 1.41 | <.001 | 1.45 | <.001 | 1.42 | <.001 | 1.39 | .01 |
| Medical condition |  |  |  |  |  |  |  |  |  |  |  |  |
| Depression | 0.92 | .44 | 0.94 | .57 | 0.87 | .21 | 0.91 | .41 | 0.91 | .37 | 0.87 | .20 |
| High blood pressure |  |  | 0.68 | <.001 |  |  | 0.67 | <.001 | 0.67 | <.001 |  |  |
| Lung disease | 0.74 | .02 | 0.77 | .05 | 0.73 | .02 | 0.77 | .05 | 0.78 | .05 | 0.75 | .03 |
| Tech Savviness |  |  |  |  |  |  |  |  |  |  |  |  |
| Social media | 0.63 | <.001 | 0.63 | <.001 | 0.63 | <.001 | 0.64 | <.001 | 0.63 | <.001 | 0.64 | <.001 |
| Internet use | 0.37 | <.001 | 0.36 | <.001 | 0.36 | <.001 | 0.36 | <.001 | 0.36 | <.001 | 0.37 | <.001 |
| Survey |  |  |  |  |  |  |  |  |  |  |  |  |
| Survey form type | 0.51 | <.001 | 0.50 | <.001 | 0.52 | <.001 | 0.49 | <.001 | 0.50 | <.001 | 0.51 | <.001 |
| Care behavior |  |  |  |  |  |  |  |  |  |  |  |  |
| Self-care ability | 0.90 | .01 | 0.88 | <.001 | 0.90 | .01 | 0.88 | <.001 | 0.89 | .01 | 0.90 | .02 |
| Care frequency | 0.64 | <.001 | 0.63 | <.001 | 0.63 | <.001 | 0.63 | <.001 | 0.63 | <.001 | 0.63 | <.001 |
| Age group 50-64 |  |  |  |  |  |  |  |  |  |  |  |  |
| Non-Hispanic other |  |  |  |  | 0.31 | <.001 | 0.30 | <.001 | 0.75 | .10 |  |  |
| Care frequency | 0.97 | .27 | 0.99 | .69 | 0.98 | .47 | 1.00 | <.001 | 0.99 | .73 | 1.02 | .53 |
| Age group ≥65 |  |  |  |  |  |  |  |  |  |  |  |  |
| Male | 0.72 | .06 | 0.75 | .10 | 0.74 | .09 | 0.71 | .04 |  |  | 0.71 | .07 |
| Less than high school |  |  | 0.63 | .09 | 0.63 | .10 | 0.71 | .22 | 0.69 | .18 | 0.73 | .25 |
| Some college |  |  |  |  | 0.70 | .03 |  |  |  |  | 0.60 | <.001 |
| Survey form type | 0.62 | .01 | 0.63 | .01 | 0.60 | <.001 | 0.65 | .01 | 0.65 | .01 | 0.60 | .01 |
| Care frequency | 1.16 | <.001 | 1.21 | <.001 | 1.20 | <.001 | 1.22 | <.001 | 1.20 | <.001 | 1.16 | <.001 |

Table S8 Sensitivity analysis including all variables except survey form type for patient portal.

|  | Original | | Excluding form type | |
| --- | --- | --- | --- | --- |
|  | Coefficient | *P* value | Coefficient | *P* value |
| Intercept | -0.44 | <.001 | -0.35 | .001 |
| Telehealth |  |  |  |  |
| Sync telehealth | 0.42 | <.001 | 0.44 | <.001 |
| Demo and SES |  |  |  |  |
| Male | -0.21 | <.001 | -0.19 | <.001 |
| Hispanic | -0.24 | <.001 | -0.28 | <.001 |
| High school graduate | -0.40 | <.001 | -0.44 | <.001 |
| Less than high school | -0.57 | <.001 | -0.55 | <.001 |
| Some college | -0.19 | <.001 | -0.20 | <.001 |
| Medium or small metro | -0.12 | .008 | -0.13 | .002 |
| Non-metro | -0.16 | .005 | -0.20 | .001 |
| Total household ≥2 | 0.11 | .011 | 0.17 | <.001 |
| Medical condition |  |  |  |  |
| Depression | 0.01 | .844 | 0.04 | .430 |
| Tech Savviness |  |  |  |  |
| Social media | 0.06 | .021 | 0.06 | .013 |
| Internet use | 0.58 | <.001 | 0.70 | <.001 |
| Survey |  |  |  |  |
| Survey form type | 0.29 | <.001 | Excluded |  |
| Care behavior |  |  |  |  |
| Self-care ability | 0.08 | <.001 | 0.08 | <.001 |
| Care frequency | 0.27 | <.001 | 0.27 | <.001 |
| Age group 50-64 |  |  |  |  |
| Sync telehealth | -0.02 | .823 |  |  |
| Non-Hispanic Black or African American | -0.26 | <.001 | -0.29 | <.001 |
| Hispanic | 0.07 | .526 |  |  |
| Less than high school |  |  | -0.18 | .177 |
| Age group ≥65 |  |  |  |  |
| Non-Hispanic Black or African American | -0.30 | <.001 | -0.35 | <.001 |
| Hispanic |  |  | 0.06 | .625 |
| Survey form type | 0.32 | <.001 |  |  |
| Care frequency | -0.07 | <.001 | -0.06 | <.001 |

Table S9 Sensitivity analysis including all variables except survey form type for synchronous telehealth.

|  | Original | | Excluding form type | |
| --- | --- | --- | --- | --- |
|  | OR | *P* | OR | *P* |
| (Intercept) | 0.10 | <.001 | 0.10 | <.001 |
| Telehealth |  |  |  |  |
| Patient Portal | 1.35 | <.001 | 1.36 | <.001 |
| Demo and SES |  |  |  |  |
| Male | 0.90 | .10 | 0.90 | .097 |
| Hispanic | 1.30 | .003 | 1.22 | .02 |
| High school graduate | 0.80 | .01 |  |  |
| Less than high school | 0.78 | .08 | 0.88 | .189 |
| Medium or small metro | 0.77 | .01 | 0.77 | .007 |
| Non-metro | 0.75 | .01 | 0.75 | .006 |
| Medical condition |  |  |  |  |
| Depression | 1.98 | <.001 | 1.93 | <.001 |
| Diabetes | 1.29 | .001 | 1.25 | .003 |
| Tech Savviness |  |  |  |  |
| Social media | 1.37 | <.001 | 1.37 | <.001 |
| Care behavior |  |  |  |  |
| Care frequency | 1.24 | <.001 | 1.24 | <.001 |
| Age group ≥65 |  |  |  |  |
| Patient Portal | 0.96 | .21 | 0.95 | .19 |
| Non-metro | 0.78 | .12 | 0.76 | .11 |

Table S10 Sensitivity analysis including all variables except survey form type for the Niether outcome.

|  | Original | | Excluding Form Type | |
| --- | --- | --- | --- | --- |
|  | OR | *P* | OR | *P* |
| (Intercept) | 8.21 | <.001 | 6.11 | <.001 |
| Demo and SES |  |  |  |  |
| Male | 1.85 | <.001 | 1.75 | <.001 |
| Hispanic | 1.32 | .018 | 1.44 | .002 |
| Total household ≥2 | 0.72 | .001 | 0.68 | <.001 |
| High school graduate | 2.69 | <.001 | 2.94 | <.001 |
| Less than high school | 2.92 | <.001 | 3.44 | <.001 |
| Some college | 1.73 | <.001 | 1.85 | <.001 |
| Large fringe metro | 0.78 | .054 | 0.77 | .043 |
| Medium or small metro | 1.28 | .011 | 1.31 | .004 |
| Non-metro | 1.39 | .005 | 1.50 | <.001 |
| Medical condition |  |  |  |  |
| Depression | 0.92 | .443 | 0.88 | .256 |
| Lung disease | 0.74 | .024 | 0.74 | .023 |
| Tech Savviness |  |  |  |  |
| Social media | 0.63 | <.001 | 0.62 | <.001 |
| Internet use | 0.37 | <.001 | 0.30 | <.001 |
| Survey |  |  |  |  |
| Survey form type | 0.51 | <.001 | Excluded |  |
| Care behavior |  |  |  |  |
| Self-care ability | 0.90 | .013 | 0.90 | .015 |
| Care frequency | 0.64 | <.001 | 0.63 | <.001 |
| Age group 50-64 |  |  |  |  |
| Non-Hispanic other |  |  | 0.46 | .049 |
| Care frequency | 0.97 | .267 | 1.01 | .727 |
| Age group ≥65 |  |  |  |  |
| Male | 0.72 | .063 | 0.74 | .052 |
| Less than high school |  |  | 0.70 | .224 |
| Survey form type | 0.62 | .006 |  |  |
| Care frequency | 1.16 | <.001 | 1.19 | <.001 |

Table S11 Sensitivity analysis using the binary patient portal outcome.

|  | Original | | Binary | |
| --- | --- | --- | --- | --- |
|  | Coefficient | *P* value | Coefficient | *P* value |
| Intercept | -0.44 | <.001 | 0.83 | <.001 |
| Telehealth |  |  |  |  |
| Sync telehealth | 0.42 | <.001 | 1.15 | <.001 |
| Demo and SES |  |  |  |  |
| Male | -0.21 | <.001 | 0.95 | .002 |
| Hispanic | -0.24 | <.001 | 0.94 | .007 |
| High school graduate | -0.40 | <.001 | 0.87 | <.001 |
| Less than high school | -0.57 | <.001 | 0.86 | <.001 |
| Some college | -0.19 | <.001 | 0.94 | <.001 |
| Medium or small metro | -0.12 | .008 | 0.96 | .009 |
| Non-metro | -0.16 | .005 |  |  |
| Total household ≥2 | 0.11 | .011 | 1.04 | .012 |
| Medical condition |  |  |  |  |
| Depression | 0.01 | .844 |  |  |
| High blood pressure |  |  | 1.04 | .016 |
| Tech Savviness |  |  |  |  |
| Social media | 0.06 | .021 | 1.02 | .057 |
| Internet use | 0.58 | <.001 | 1.21 | <.001 |
| Survey |  |  |  |  |
| Survey form type | 0.29 | <.001 | 1.07 | .001 |
| Care behavior |  |  |  |  |
| Self-care ability | 0.08 | <.001 | 1.03 | .004 |
| Care frequency | 0.27 | <.001 | 1.09 | <.001 |
| Age group 50-64 |  |  |  |  |
| Sync telehealth | -0.02 | .823 |  |  |
| Non-Hispanic Black or African American | -0.26 | <.001 |  |  |
| Hispanic | 0.07 | .526 | 1.01 | .810 |
| Less than high school |  |  | 0.92 | .092 |
| Age group ≥65 |  |  |  |  |
| Sync telehealth |  |  | 0.99 | .825 |
| Non-Hispanic Black or African American | -0.30 | <.001 | 0.93 | .003 |
| Hispanic |  |  | 0.99 | .681 |
| Non-metro |  |  | 0.94 | .022 |
| Survey form type | 0.32 | <.001 | 1.13 | <.001 |
| Care frequency | -0.07 | <.001 | 0.98 | <.001 |

Table S12 Use of patient portals and synchronous telehealth across the survey cycles

|  |  | Patient portal use (n = 12865, 100.0 %) | | | | | Synchronous telehealth use (n = 12865, 100.0 %) | |
| --- | --- | --- | --- | --- | --- | --- | --- | --- |
|  |  | 0 (n = 4482, 34.8%) | 1 to 2 times (n = 2590, 20.1%) | 3 to 5 times (n = 2605, 20.2%) | 6 to 9 times (1382, n = 10.7%) | 10 or more times (1806, n = 14.0%) | No use (n = 7923, 61.6%) | Use (n = 4942, 38.4%) |
| **Cycle (n = 12865, 100.0 %) n, row-wise %** | |  |  |  |  |  |  |  |
|  | Cycle 6  (n = 5873, 45.7%) | 2253 (38.4) | 1185 (20.2) | 1118 (19) | 575 (9.8) | 742 (12.6) | 3410 (58.1) | 2463 (41.9) |
|  | Cycle 7  (n = 6992, 54.3%) | 2229 (31.9) | 1405 (20.1) | 1487 (21.3) | 807 (11.5) | 1064 (15.2) | 4513 (64.5) | 2479 (35.5) |
